# The association between accelerometer-assessed physical activity and respiratory function in older adults differs between smokers and non-smokers

**DOI:** 10.1038/s41598-019-46771-y

**Published:** 2019-07-16

**Authors:** Mohamed Amine Benadjaoud, Mehdi Menai, Vincent T. van Hees, Vadim Zipunnikov, Jean-Philippe Regnaux, Mika Kivimäki, Archana Singh-Manoux, Séverine Sabia

**Affiliations:** 1 Institute for Radiological Protection and Nuclear Safety (IRSN), Fontenay-Aux-Roses, France; 2 Inserm U1153, CRESS, Epidemiology of Ageing and Neurodegenerative diseases, Université de Paris, Paris, France; 3 Netherlands eScience Center, Amsterdam, The Netherlands; 4 Department of Biostatistics, Johns Hopkins Bloomberg School of Public Health, Baltimore, 21205 USA; 5 EHESP, Center of Research in Epidemiology and Statistics - UMR 1153, F-35000 Rennes, France; 6 Department of Epidemiology and Public Health, University College London, London, United Kingdom

**Keywords:** Signs and symptoms, Risk factors

## Abstract

The association between physical activity and lung function is thought to depend on smoking history but most previous research uses self-reported measures of physical activity. This cross-sectional study investigates whether the association between accelerometer-derived physical activity and lung function in older adults differs by smoking history. The sample comprised 3063 participants (age = 60–83 years) who wore an accelerometer during 9 days and undertook respiratory function tests. Forced vital capacity (FVC) was associated with moderate-to-vigorous physical activity (MVPA; acceleration ≥0.1 *g* (gravity)) in smokers but not in never smokers: FVC differences for 10 min increase in MVPA were 58.6 (95% Confidence interval: 21.1, 96.1), 27.8 (4.9, 50.7), 16.6 (7.9, 25.4), 2.8 (−5.2, 10.7) ml in current, recent ex-, long-term ex-, and never-smokers, respectively. A similar trend was observed for forced expiratory volume in 1 second. Functional data analysis, a threshold-free approach using the entire accelerometry distribution, showed an association between physical activity and lung function in all smoking groups, with stronger association in current and recent ex-smokers than in long-term ex- and never-smokers; the associations were evident in never smokers only at activity levels above the conventional 0.1 *g* MVPA threshold. These findings suggest that the association between lung function and physical activity in older adults is more pronounced in smokers than non-smokers.

## Introduction

Poor lung function, characterized by low forced expiratory volume in 1 second (FEV_1_) and low forced vital capacity (FVC), is associated with an increased risk of death^1,2^ and chronic conditions, such as lung cancer^3^ and cardiovascular diseases.^4,5^ Lung capacity is largely determined by endogenous factors such as age, sex, and body size and early life exposures^6,7^. However, there is growing interest in modifiable risk factors for poor lung function and so far only the role of smoking is widely recognized^8^. The beneficial impact of physical activity on progression of chronic obstructive pulmonary disease (COPD) or asthma^9,10^ has led researchers to assess the role of physical activity^11–18^ and sedentary behavior^14,19^ as determinants of poor lung function in the general population. The results from these studies suggest that the association of physical activity with lung function might depend on smoking history^11–13,15,17,18^ such that physical activity potentially mitigates the adverse impact of smoking on lung function in smokers, possibly via anti-inflammatory and vascular mechanisms^20^.

Most studies on the association between physical activity and lung function rely on self-reported physical activity data which are prone to reporting bias. Physical activity assessed objectively by accelerometers is more strongly associated with health outcomes^21–23^. Typically, these studies categorize the duration of physical activities at different intensities (from sedentary behaviour to moderate-and-vigorous physical activity, MVPA) although this method represents important loss of information as the physical activity scale is a continuum. To address this issue, functional data analysis is useful in order to model the entire distribution of intensities of accelerometer data^24–26^.

The present study aims to examine whether the cross-sectional association between accelerometer-assessed physical activity and lung function differs by smoking history in a large population-based study of older adults aged 60 to 83 years. To address some of the limitations in current evidence, we use (1) usual categories of activity intensities and indicators of lung function, FEV_1_ and FVC, and (2) functional data analysis^27^ to identify activity intensity ranges associated with performance on spirometry test.

## Methods

### Study population

Data are drawn from the Whitehall II cohort study that was established in 1985/88 on 10308 British civil servants (67% men) aged 35–55 years^28^. The study design consists of a clinical examination every 4–5 years since inclusion. Accelerometer measurement was added to the study at the 2012/13 wave of data collection (age range = 60–83 years) for participants seen at the central London clinic and for those living in the South-Eastern regions of England who had their clinical assessment at home.

Participants gave informed, written consent to participate, and the University College London Hospital Committee on the Ethics of Human Research approved the study, reference number 85/0938. All experiments were performed in accordance with relevant guidelines and regulations.

### Physical activity

During the 2012/13 clinical examination, participants were asked to wear a validated^29^ triaxial accelerometer (GENEActiv; Activinsights Ltd, Kimbolton, Cambs, UK) on their non-dominant wrist for 9 consecutive, 24-hour, days. The data processing has been described elsewhere^23^. In brief, the accelerometer sampled data at 85.7 Hz rate, acceleration was expressed relative to gravity (*g* units; 1 *g* = 9.81 m.s^−2^)^30^, averaged over 5-second epochs^31–33^, and corrected for calibration error^34^.

Accelerometer data were processed in R using the GGIR package version 1.2–11 (https://cran.r-project.org/src/contrib/Archive/GGIR/). Sleep periods were detected using a validated algorithm aided by a sleep log^35^. Data from the first waking up (day 2) to waking up on the day before last day (day 8) were used, corresponding to 7 full days. Only waking periods were retained in the analysis, that is periods between waking and sleep onset (as opposed to the night period). Participants were included in the analysis if they had valid data, defined as daily wear time ≥2/3 of waking hours, for at least 2 weekdays and 2 week-end days. In those with valid data, nonwear time was corrected for using a previously reported algorithm^30,33,36^.

In order for the activity undertaken to be classified as MVPA, mean acceleration over 5s-epoch needed to be ≥0.10*g*, between 0.03*g* and 0.10*g* for light activity, and <0.03*g* for sedentary behavior^21,23,32,37^. The daily time in different activity level was calculated as the mean of measures over 7 days. For participants with <7 valid days (N = 117), data from weekend and week-days were weighted to represent a 7-day week^21–23^.

### Lung function

Lung function was measured at the clinical examination in 2012/13 without inhalation of bronchodilators using a portable flow spirometer (ndd Easy on-PC Spirometer, Zurich, Switzerland) administered by a trained nurse. Participants with health contraindications were not allowed to perform the lung function tests (details in Supplementary Methods 1). Several parameters were recorded: FEV_1_, peak expiratory flow, the 25^th^, 50^th^, and 75^th^ percentile forced expiratory flow, and FVC. FVC measures the volume of air that can forcibly be blown out after full inspiration, measured in milliliters. FEV_1_ measures the volume of air expelled in the first second during the FVC maneuver, again measured in milliliters^38^. Among the 5 attempted tests, we retained the one with the largest FEV_1_.

### Smoking history

Smoking status was assessed by questionnaire every 4–5 years since inclusion. Participants were classified based on their smoking history in 2012/13: current smokers, recent ex-smokers (smoking cessation within 10 years), long-term ex-smokers (smoking cessation more than 10 years before 2012/13) and never smokers.

### Covariates

*Height and weight* were assessed by a trained nurse during the clinical examination. Height was measured in bare feet to the nearest millimetre using a stadiometer, while the participant stood completely erect with the head in the Frankfort plane. Weight was measured in underwear to the nearest 0.1 kg using an electronic Soehnle scale with a digital readout (Leifheit AS, Nassau, Germany). *Sociodemographic variables* were assessed by questionnaire and included age, sex, ethnicity (Caucasians, non-Caucasians), marital status (married/cohabiting, other), education (5-level variable) and occupational position at age 50 years (high, intermediate or low, representing income and status at work). *Health behaviours* were assessed by questionnaire and included alcohol consumption (number of alcoholic drinks consumed in the last seven days, converted to units of alcohol consumed in a week and categorized as “no/occasional alcohol consumption”, “moderate alcohol consumption” (1–14 units/week in women, 1–21 units/week in men), and “heavy alcohol consumption” (≥14 units in women, ≥21 units in men)), and frequency of fruit and vegetables consumption. Among current and recent ex-smokers, the daily number of cigarettes smoked was self-reported. *Respiratory diseases*, including COPD and asthma, were identified using linkage to national hospital records over the follow-up (1985–2013) and self-reported information on long-standing illness in 2012/13. *The number of chronic diseases* was estimated based on records of coronary heart disease, stroke, cancer, depression, diabetes, arthritis, Parkinson’s disease, and dementia identified using linkage to national hospital records and self-reported information on long-standing illness over the follow-up (1985 to 2013).

### Statistical analyses

Two sets of analyses were conducted, described below. For all analyses, the significance level was 0.05 and all tests were two-sided.

#### Association of time spent in activity levels with FEV_1_ and FVC

We first assessed the association between physical activity and lung function in the total study population using linear regressions adjusted for age, sex, ethnicity, height, weight, smoking status, time spent in physical activity level under consideration, and waking duration (corresponding to the total time spent in sedentary, light and moderate-to-vigorous activities), and then additionally for socio-demographic and behavioural factors, respiratory disease and the number of chronic diseases. Adjustment for height and weight was preferred to adjustment for body mass index as the model fit was better for the former (Δ Akaike Information Criteria = 489 for FEV_1_ and 735 for FVC). Then, to assess whether the association differs by smoking history, we included interaction terms between time spent in activity level under consideration and smoking history (see equation in Supplementary Methods 2). No interactions were found between other covariates and smoking history (all p for interaction >0.22) leading us to not include interactions terms with other covariates in the model. In order to assess whether the associations observed were driven by presence of respiratory diseases, analyses were repeated excluding participants with respiratory disease.

#### Association between accelerometry distribution and expired air volume curve using functional data analysis

For each participant indexed by *i*, the distribution density function of the 5s-epoch acceleration intensities of the waking periods during the observation, designed by *f*_*i*_, was determined using a kernel density estimation^39^ with a Gaussian kernel and a plug-in bandwidth selector method^40^ implemented in the package “ks” of the R software (version 3.5.1 The R Foundation for Statistical Computing http://www.r-project.org/). As acceleration data are skewed, the transformation log(1 + acceleration) was applied prior to the kernel smoothing. The complete diurnal activity distribution for this subject was then represented by a single function ${A}_{i}(x)={T}_{i}\times {f}_{i}(x)$ where *T*_*i*_ represents the daily waking time and *x* is the magnitude of acceleration variable which takes its values over the range of the recorded data measured on *g* units (see interpretation in Supplementary Methods 3).

For each participant, the flow-volume curve noted F, was reconstituted using the peak expiratory flow, the 25^th^, 50^th^, and 75^th^ percentile forced expiratory flow, and FVC using penalized spline regression. The expired air volume curve was then deduced as the solution of the autonomous differential equation $\frac{d}{dt}y(t)=F(y(t))$ with the condition $y(1\,second)=FE{V}_{1}$ (see interpretation in Supplementary Methods 4). The volume-time function *y*(*t*) expresses the volume of air exhaled as a function of time (second).

The association between accelerometry distribution, *A*_*i*_(*x*), and expired air volume-time function *y*(*t*) was assessed using a function-on-function regression adjusted for covariates and expressed using regression coefficient surfaces (see equation in Supplementary Methods 5). This approach allows the identification of the range of accelerometry associated with the air volume expired at specific times of the spirometry test. Due to the complexity of the method, the number of covariates included was limited to age, sex, ethnicity, height, weight, respiratory disease, and number of chronic diseases^6,7^. The function-on-function regression was undertaken using the REFUND package in R (https://cran.r-project.org/web/packages/refund/refund.pdf). This method allowed us to identify accelerometry intensity threshold above which physical activity is associated with better lung function in each smoking history group. Then the association of time spent above this threshold with FEV_1_ and FVC was estimated (see details in Supplementary Methods 6).

## Results

**Figure 1:**
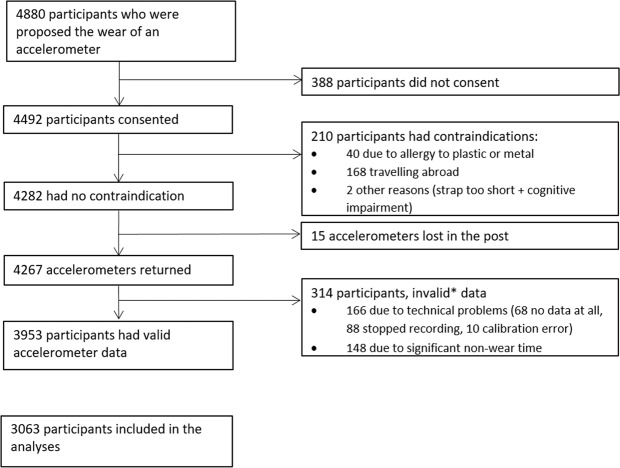
Flow Chart of the Study. * valid data defined as daily wear time ≥2/3 of waking hours, for at least 2 weekdays and 2 week-end days.

Among the 4880 participants to whom the accelerometer assessment was proposed, 388 did not consent, 210 had contraindications, 15 had their accelerometer lost in the post, 314 did not have valid accelerometer data, and 890 did not have all data points from the spirometry test to rebuild their volume-time curve (flow chart in Fig. 1). Compared to participants not included in the analysis (N = 1817), the analytic sample (N = 3063) did not differ by age (69.2 (standard deviation (SD)=5.6) vs. 69.0 y (SD=5.6), *P*=0.20), but was composed of more men (73.9% vs. 65.3%, *P* < 0.0001) and fewer participants from the lowest occupational position (11.0% vs. 12.4%, *P* = 0.03). Among the 3,063 participants included in the analysis, 2,946 (96.2%) had valid accelerometer data for 7 days, 76 (2.5%) for 6 days, and 41 (1.3%) for 4–5 days. In total missing data were replaced for 1–2 hours over the full observational period for 24.5% of the participants, 2–5 hours for 1.0% of the participants, 5–10 hours for 0.6% of the participants, and 10–25 hours for 0.1% of the participants. Table 1 presents characteristics of the study population.

**Table 1:** Characteristics of the study population.

	All (n = 3063)	Smoking history				p
		Current smokers (n = 86)	Recent ex-smokers (n=146)	Long-term ex-smokers (n = 1251)	Never smokers (n = 1580)	
	N = 3063	N = 86	N = 146	N = 1251	N = 1580	
Age (years), M(SD)	69.2 (5.6)	67.3 (4.8)	68.2 (5.2)	69.7 (5.6)	69.0 (5.7)	<0.001
Men (%)	73.9	69.8	75.3	77.9	70.8	<0.001
Caucasian (%)	93.4	93.0	94.5	95.5	91.7	0.003
Height (cm), M(SD)	170.8 (9.1)	170.3 (8.4)	170.7 (8.6)	171.3 (8.7)	170.5 (9.4)	0.08
Weight (kg), M(SD)	77.7 (14.1)	78.4 (16.2)	80.0 (14.5)	79.0 (14.3)	76.4 (13.8)	<0.001
High occupational position at age 50y (%)	45.5	27.9	39.7	45.1	47.3	0.002
Higher education than university (%)	31.4	19.8	21.9	27.3	36.1	<0.001
Married/cohabitating (%)	75.7	58.1	69.9	79.0	74.7	<0.001
High alcohol consumption (%)	14.4	24.4	25.3	18.6	8.7	<0.001
Daily fruit and vegetable consumption (%)	80.3	54.7	75.9	79.1	83.1	<0.001
Cigarettes smoked (per day), M(SD)		12.9 (7.7)	5.6 (11.1)*			<0.001
Respiratory diseases (%)	8.0	7.0	15.1	7.9	7.5	0.01
One or more chronic diseases (%)	46.7	48.8	54.1	51.1	42.5	<0.001
**Time spent per day** (**min**), **M**(**SD**)						
Sedentary behaviour	673.9 (88.1)	698.4 (76.8)	674.4 (105.6)	671.9 (86.1)	674.1 (88.4)	0.03
Light physical activity	240.5 (58.1)	220.8 (52.9)	237.3 (70.8)	240.8 (56.6)	241.7 (58.2)	0.009
MVPA	69.3 (36.7)	58.0 (31.3)	69.2 (41.2)	70.1 (37.0)	69.3 (36.2)	0.05
FVC (ml), M(SD)	3639.4 (925.4)	3407.9 (989.9)	3462.4 (973.9)	3667.2 (887.6)	3646.5 (943.7)	0.008
FEV_1_ (ml), M(SD)	2790.5 (742.2)	2501.1 (767.4)	2565.9 (816.0)	2809.5 (713.1)	2811.8 (750.2)	<0.001

Abbreviations: FEV_1,_ Forced Expiratory Volume in 1 sec; FVC, Forced Vital Capacity; MVPA, Moderate to vigorous physical activity. p: Chi-Square or Anova.

^*^Number of cigarettes smoked before they stopped smoking.

In models adjusted for age, sex, ethnicity, height and weight, compared to never smokers, current, recent, and long-term ex-smokers had respectively 360.0 (95%CI = 254.1, 466.0), 305.6 (95%CI = 221.8, 387.5) and 55.0 (18.5, 91.5) ml lower FEV_1_, and 278.6 (95%CI = 157.2, 400.0), 238.3 (95%CI = 143.3, 333.2) and 47.6 (95%CI = 5.7, 89.4) ml lower FVC (data not tabulated).

### Association of time spent in activity levels with FEV_1_ and FVC

In analyses adjusted for age, sex, ethnicity, height, weight, and waking duration, the interaction terms between time spent in MVPA and smoking history (entered as an ordinal variable) were significant for both FEV_1_ (p = 0.0002) and FVC (p < 0.0001). In fully adjusted analyses, 10 minutes greater MVPA was associated with 58.6 (95%CI = 21.1, 96.1) ml higher in FVC in current smokers, 27.8 (95%CI = 4.9, 50.7) ml increase in recent ex-smokers, 16.6 (95%CI = 7.9, 25.4) ml in long-term ex-smokers, and only 2.8 (95%CI = −5.2, 10.7) ml in never smokers (Table 2). Similarly, the association between sedentary time and lung function was evident in current but not in never smokers (p for interaction = 0.02 for FEV_1_ and 0.03 for FVC). There was no evidence of an association of FEV_1_ and FVC with light physical activity in all smoking groups, the p for interaction did not reach significance for FEV_1_ (p = 0.06) and FVC (p = 0.09). After exclusion of participants with respiratory disease, the association of MVPA with both FEV_1_ and FVC was slightly attenuated whereas the association with sedentary time remained only for FVC among current smokers (Supplementary Table 1).

**Table 2:** Association between physical activity and respiratory function.

	Total population	Smoking history			
		Current smokers	Recent ex-smokers	Long-term ex-smokers	Never smokers
	β* [95% CI]	β [95% CI]	β [95% CI]	β [95% CI]	β [95% CI]
**FVC** (**ml**)					
Model 1^†^					
For 10 min increase per day					
Sedentary behaviour	−2.5 [−5.0, −0.0]	−19.2 [−34.7, −3.7]	−6.1 [−15.0, 2.8]	−2.8 [−6.6, 0.9]	−1.2 [−4.4, 2.1]
Light activity	0.8 [−2.7, 4.4]	14.1 [−8.4, 36.6]	0.4 [−13.0, 13.7]	3.6 [−2.0, 9.2]	−1.7 [−6.6, 3.1]
MVPA	11.9 [5.9, 17.9]	60.3 [22.3, 98.2]	33.8 [11.1, 56.6]	16.6 [7.8, 25.4]	3.6 [−4.4, 11.6]
Model 2^‡^					
For 10 min increase per day					
Sedentary behaviour	−2.6 [−5.1, −0.1]	−19.4 [−34.7, −4.1]	−4.0 [−12.9, 4.9]	−3.3 [−7.1, −0.5]	−1.1 [−4.4, 2.1]
Light activity	1.2 [−2.4, 4.8]	14.0 [−8.3, 36.3]	−0.4 [−13.6, 12.8]	4.1 [−1.4, 9.6]	−1.5 [−6.3, 3.4]
MVPA	11.4 [5.4, 17.4]	58.6 [21.1, 96.1]	27.8 [4.9, 50.7]	16.6 [7.9, 25.4]	2.8 [−5.2, 10.7]
**FEV**_**1**_ (**ml**)					
Model 1^†^					
For 10 min increase per day					
Sedentary behaviour	−1.0 [−3.2, 1.2]	−13.2 [−26.7, 0.4]	−6.5 [−14.2, 1.3]	−1.0 [−4.6, 2.3]	0.2 [−2.7, 3.0]
Light activity	−0.7 [−3.8, 2.5]	9.4 [−10.2, 29.1]	3.3 [−8.3, 15.0]	0.9 [−4.0, 5.7]	−2.7 [−7.0, 1.5]
MVPA	7.7 [2.4, 12.9]	32.6 [−0.5, 65.7]	31.5 [11.7, 51.4]	11.8 [4.1, 19.4]	0.5 [−6.5, 7.5]
Model 2^**‡**^					
For 10 min increase per day					
Sedentary behaviour	−1.1 [−3.3, 1.0]	−13.8 [−26.9, −0.7]	−4.8 [−12.4, 2.8]	−1.6 [−4.8, 1.6]	0.2 [−2.5, 3.0]
Light activity	−0.2 [−3.2, 2.9]	8.6 [−10.5, 27.6]	3.8 [−7.6, 15.1]	1.6 [−3.1, 6.3]	−2.5 [−6.6, 1.6]
MVPA	7.5 [2.3, 12.6]	31.2 [−0.9, 63.3]	27.6 [7.9, 47.2]	12.3 [4.8, 19.8]	−0.2 [−7.0, 6.6]

Abbreviations: CI, confidence interval; FEV_1,_ Forced Expiratory Volume in 1 sec; FVC, Forced Vital Capacity; MVPA, Moderate to vigorous physical activity.

*Additional adjustment for smoking history.

^†^Model 1: adjusted for age, sex, ethnicity, height, weight, and waking duration.

^**‡**^Model 2: Model 1 additionally adjusted for occupational position at age 50y, education, marital status, alcohol consumption, fruit and vegetable consumption, respiratory disease, and number of chronic diseases. Additionally adjusted for number of cigarettes smoked per day among the current and recent ex-smokers (corresponding to cigarettes smoked before they quitted smoking) groups.

### Association between diurnal accelerometry distribution and expired air-volume curve using functional data analysis

**Figure 2:**
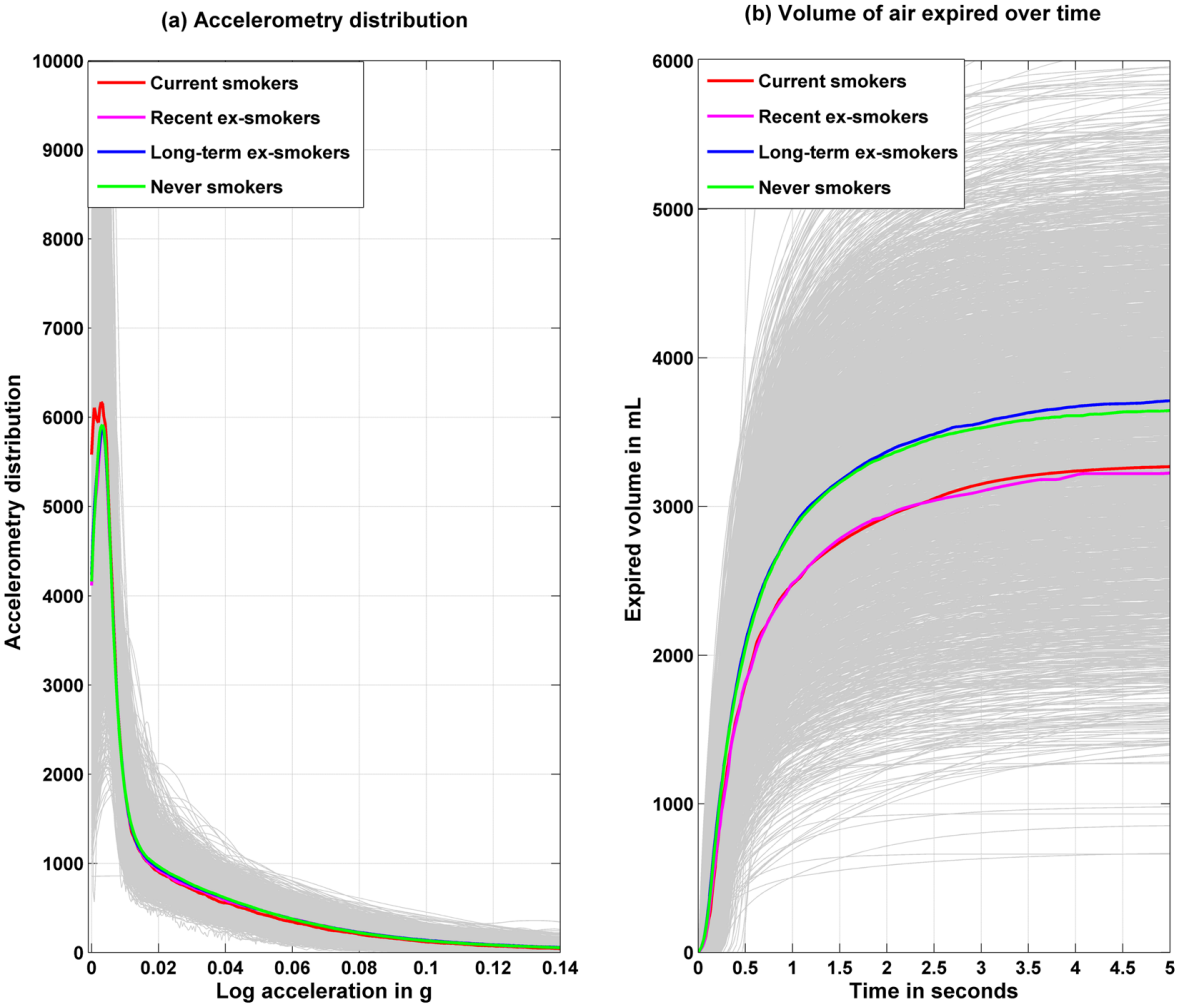
Accelerometry distribution during waking time and volume of air expired over time as a function of smoking status. Grey curves represent individual daily accelerometry distribution over waking time (left panel, number of 5s-epoch over diurnal time) and expired air volume over the time of the spirometry test (right panel). Colored curves represent the median curves among the different smoking history groups.

The accelerometry distribution *A*_*i*_(*x*) and the expired air volume-time curve *y*_*i*_(*t*) are plotted in Fig. 2; current smokers spent more time in the lowest accelerometry range. Differences in spirometry test were evident all along the distribution, with never and long-term ex-smokers having better lung function profiles. The plot of the expired air volume-time curve are displayed in the top row of Supplementary Fig. 1 for all the participants, with median functions separately in men and women (left panel), age quartiles (middle panel) and in Caucasians and non-Caucasians (right panel). The regression coefficients for the association of sex, age (per 1 year) and ethnicity with the expired air volume are shown with their 95% confidence interval in the bottom row. These results show the association of these covariates along the continuum of spirometry performance and not confined to 1 second (FEV_1_) or at 5 seconds (FVC) as used in the classical multivariate approach. The direction and strength of association using functional coefficients are consistent with results from the multivariate regression for FEV_1_ (beta for women vs men = −474.7 (−527.8, −421.5) ml) and FVC (beta for women vs men = −576.3 (−638.0, −514.7) ml) at 1 second and 5 second respectively.

**Figure 3:**
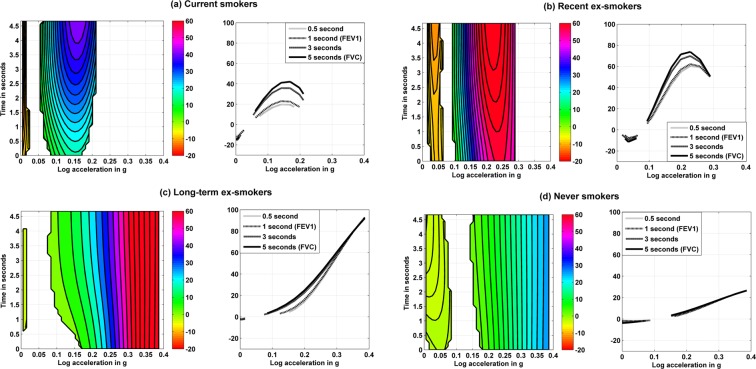
Coefficient surfaces representing the association between accelerometry distribution (X axis) and volume of expired air over time (Y axis) and their slices at times 0.5, 1 (FEV_1_), 3, and 5 (FEC) seconds by smoking history*. *Coefficient surfaces and their slices at times 0.5, 1 (FEV_1_), 3, and 5 (FVC) seconds (only significant coefficients at P < 0.05 are shown), from a function-to-function regression model to assess the association between expired air volume-time curve (Y axis) and accelerometry distribution (X axis) adjusted for age, sex, ethnicity, height, weight, respiratory disease, number of chronic diseases, and accelerometry distribution by smoking history groups. Positive values indicate that more time spent in a given accelerometry range is associated with higher air expired volume over the spirometry time scale whereas negative values indicate that more time spent in a given accelerometry range is associated with lower air expired volume over the spirometry time scale.

Results from function-on-function regression model are presented in Fig. 3, showing the significant coefficient surfaces in each smoking history group and their slices at times 0.5, 1 (FEV_1_), 3, and 5 (FVC) seconds. The acceleration levels above which association with spirometry measure was evident were higher in a graded fashion from current to never-smoker groups. A positive (protective) association between physical activity and FVC (time = 5 seconds) was observed for accelerometry values above 0.054*g* among current smokers, 0.094*g* among recent ex-smokers, 0.074*g* among long-term ex-smokers and 0.161*g* among never smokers (Table 3). More time spent in activity intensities above these smoking-specific accelerometry thresholds was associated with higher FVC, with stronger associations observed in current and recent ex-smokers (Table 3). A similar trend was observed for FEV_1_. Results in the lower range of accelerometry were less consistent but overall lower lung function was observed for more time spent at lower accelerometry level among current smokers (<0.03*g*).

**Table 3:** Association between physical activity and respiratory function using threshold for benefits identified using functional data analysis.

	Current smokers	Recent ex-smokers	Long-term ex-smokers	Never smokers
**FVC** (**ml**)				
Accelerometry threshold	0.054	0.094	0.074	0.161
${\rm{\Delta }}FVC$ *[95% CI] for 10 min increase per day in activity intensity above the threshold	34.7 [23.3, 49.1]	36.5 [29.4, 47.4]	15.1 [13.1, 22.4]	13.2 [7.5, 20.1]
**FEV**_**1**_ (**ml**)				
Accelerometry threshold	0.060	0.093	0.122	0.150
${\rm{\Delta }}FE{V}_{1}$ *[95% CI] for 10 min increase per day in activity intensity above the threshold	21.5 [15.1, 28.7]	30.2 [23.7, 35.9]	11.0 [8.5, 13.7]	8.9 [5.3, 12.8]

Abbreviations: CI, confidence interval; FEV_1,_ Forced Expiratory Volume in 1 sec; FVC, Forced Vital Capacity; CI, confidence interval.

*Estimated change from a function-to-function regression model adjusted for age, sex, ethnicity, height, weight, respiratory disease, and number of chronic diseases.

## Discussion

### Principal findings

This study of 3063 adults aged 60 to 83 years presents three key findings. First, results based on conventional categorization of physical activity showed that the association between MVPA and lung function is dependent on smoking history, with associations being evident only in current and ex-smokers. Second, function-on-function regression, which takes the continuum of measurement into account, shows an association between physical activity and lung function, both FEV_1_ and FVC, in all smoking groups but only at very high levels of physical activity in never smokers. Third, lung function is less consistently associated with time spent in sedentary activity, the association being only evident for FVC among current smokers.

### Comparison with others studies

Longitudinal studies based on self-reported physical activity measures have suggested that physical activity in midlife and early old age is associated with slower decline in lung function^16,17,41^. In studies that have investigated the modifying role of smoking, physical activity was found to be associated with higher FEV_1_ and FVC^12^, slower decline in lung function^13^ and lower risk of incident chronic obstructive pulmonary disease^11^, only in current or ever-smokers. One study using accelerometer-assessed physical activity among 341 adults assessed the cross-sectional association of MVPA with lung function, without accounting for time spent in sedentary behavior and light activities, and also reported the association with MVPA to be evident only in smokers^15^. Our study using objective physical activity data on a large sample of older adults adds to the previous findings by showing that the activity intensity threshold at which there is an association between physical activity and lung function depends on smoking history. The association between spirometry measure and physical activity was evident starting from activities in the higher range of light intensities (acceleration ≥0.06*g*) in smokers, from physical activities of moderate-to-vigorous intensities (acceleration ≥0.10*g*) in ex-smokers and from even more intense MVPA in never smokers. The associations were found all along the spirometry measure, including both FEV_1_ and FVC. Several mechanisms could underlie the association between physical activity and lung function. They may involve the anti-inflammatory and vascular benefits of physical activity^20^. These mechanisms might play a more pronounced role among smokers by compensating for the deleterious effect of smoking on the lung^13,20^. As the study is cross-sectional, the observed association could also reflect the inability of those with poor lung function to perform physical activity at higher intensity and this would be more pronounced among smokers who might carry multiple health conditions affecting their ability to undertake more intense physical activity^42^.

Sedentary behaviour is thought to be deleterious for health^43^. However, it is unclear whether time spent sedentary is important because of the adverse effects of inactivity or because it reduces the time available for activities at more intense levels^43^, given that a day is constrained naturally to 24 hours. Few studies have assessed the impact of sedentary behaviour on lung function and no robust association has been reported^14,19^. In the present study, an association between spirometry measure and sedentary time was found only in current smokers. In other smoking groups, the association was small and inconsistent across the various analytic approaches. Both the classical and functional approaches accounted for the diurnal duration constraint so that increase in sedentary time corresponds to decrease in active time. Our findings of an association between sedentary behaviour and lung function in current smokers could reflect an aggravating effect of sedentary behaviour on lung function in smokers or the likelihood of smokers with poor lung function to be more sedentary.

### Strengths and limitations

Strengths of this study include the large sample size, use of accelerometer-assessed physical activity, and functional data analysis approach that is free from laboratory-based thresholds definition of sedentary, light and moderate-to-vigorous activity and accounts for the entire spirometry and diurnal activity intensity distribution. We were able to identify intensities at which physical activity was associated with respiratory function in groups defined by smoking history. The limitations of this study include its cross-sectional design, although sensitivity analysis excluding COPD and asthma cases were conducted to assess whether the association observed between physical activity and spirometry measure was not driven by difficulties in undertaking physical activity among those with respiratory diseases, and the results were broadly similar. Further longitudinal studies are needed to assess the direction of the association between physical activity and respiratory function. Second, although the number of current smokers was small, the trend observed across the smoking groups suggested that the results in smokers were not likely to be by chance. However, future studies are needed to confirm effect size in this group. Finally, although wrist-mounted accelerometer are not designed to distinguish between sitting and inactive standing position, the 0.03 g threshold we used has been reported to accurately separate sedentary behaviours from common motion-based light-intensity activities^37^.

## Conclusion

This study on older adults showed an association between physical activity and better lung function, particularly in recent ex- and current smokers. Among never smokers, the intensity of physical activity required to create an association with lung function was well above that used to define MVPA. These findings suggest that the association between physical activity and lung function might have been underestimated in non-smokers in previous studies that did not differentiate between intensities of MVPA.

## Supplementary information

Supplementary materials

## Data Availability

Whitehall II data, protocols, and other metadata are available to the scientific community. Please refer to the Whitehall II data sharing policy at https://www.ucl.ac.uk/whitehallII/data-sharing.
