# Supplementary material for: The association between accelerometer-assessed physical activity and respiratory function in older adults differs between smokers and non-smokers
Source: Sci Rep. 2019 Jul 16;9:10270. doi: 10.1038/s41598-019-46771-y (PMC6635399; doi:10.1038/s41598-019-46771-y)
Supplement: Supplementary file 1 — Supplementary materials [file 41598_2019_46771_MOESM1_ESM.pdf]

SUPPLEMENTARY INFORMATION

The association between accelerometer-assessed physical activity and respiratory function in older adults differs between smokers and non-smokers

Mohamed Amine Benadjaoud,[1]* Mehdi Menai,[2*] Vincent T. van Hees,[3] Vadim Zipunnikov,[4] Jean-Philippe Regnaux,[5] Mika Kivimäki,[6] Archana Singh-Manoux,[2,6] Séverine Sabia[2,6]

* These authors contributed equally.

Supplementary Methods 1. Lung function contraindications

Participants with the following conditions were not allowed to perform the lung function tests: persons who had recently been coughing up blood of unknown origin, ever had a pneumothorax, had recently an embolism, had recently a heart attack or stroke, had be admitted to hospital with a heart complaint in the last month, had ever been diagnosed with an aneurysm in chest, brain or stomach, had recently undergone ear or eye surgery, had recently undergone stomach or chest surgery, had recently a detached retina or had medication for tuberculosis.

Supplementary Methods 2. Linear regression model with interaction terms

Equation

$$\text{FVC or FEV}_1 = \alpha_0 + \alpha_S \times D_S + \alpha_{RES} \times D_{RES} + \alpha_{LES} \times D_{LES}$$

$$+\beta_0 \times X_{PA} + \beta_S \times D_S \times X_{PA} + \beta_{RES} \times D_{RES} \times X_{PA} + \beta_{LES} \times D_{LES} \times X_{PA}$$

$$+\sum_l \gamma_l \times Covariates_l \qquad (1)$$

where $D_S, D_{RES}\ and\ D_{LES}$ are “dummy” binary variables indicating the smoking history category as follows: D_S, for smokers, D_{RES} for recent ex-smokers, and D_{LES} for long-term ex-smokers. X_{PA} represents time spent in the activity level of interest and $Covariates_l$ the covariates included in the model. The interaction regression model was preferred to a stratified approach as it allows to test whether smoking history is a modifying factor for the association with physical activity according to the statistical significance of the interaction parameter estimates $\beta_S, \beta_{RES}\ and\ \beta_{LES}$. In addition, as there was no significant interaction between smoking status and other covariates, this approach has the advantage of having a unique estimate for each of covariates rather than one in each smoking group as it would be the case in stratified analyses. The parameters $\alpha_0\ and\ \beta_0$ represent the intercept and the physical activity coefficient for the never smokers category which is considered as a reference category. α_S, α_{RES}, $and\ \alpha_{LES}$ represent difference in lung function according to smoking status (intercept).

Supplementary Methods 3. Interpretation of the accelerometry distribution function

Several parameters can be estimated from the daily accelerometry distribution function $A_i(x)$. For example, the daily duration of the MVPA for the ith subject, noted X_i^{MVPA}, can be calculated as

$$X_i^{MVPA} = \int_{0.10}^{+\infty} A_i(x)dx = T_i \times \int_{0.10}^{+\infty} f_i(x)dx$$

where T_i represents the daily waking time and x is a variable which takes its values over the range of the recorded data measured on g units. Indeed, by construction of the probability density function $f_i(x)$, the integration term $\int_{0.10}^{+\infty} f_i(x)dx$ represents the proportion of the daily waking time where acceleration is greater than 0.10g.

Supplementary Methods 4. Interpretation of the expired air volume-time curve

The parameters FEV_1 and FVC can be estimated from the functional object $y(t)$ and equal to the values $y(1\ second)$ and $y(5\ seconds)$ respectively, with the assumption that expiration lasts a maximum of 5 seconds (12 breaths/minute). The right panel of Figure 1 shows the expired air volume-time curves for all participants as well as medians in each smoking groups. The median dynamics show a similar pattern for the never smokers and long term ex-smokers in on hand, and for the current and recent ex-smokers in the other hand.

Supplementary Methods 5. Function-to-function regression

This model is similar to the interaction regression model (equation 1) previously formulated apart that the outcome is now a function of time (expired air volume-time function) and physical activity is the function of accelerometry distribution:

Equation

$$y_i(t) = \alpha_{NS}(t) \times D_{NS} + \alpha_S(t) \times D_S + \alpha_{RES}(t) \times D_{RES} + \alpha_{LES}(t) \times D_{LES}$$

$$+ \int \beta_{NS}(t,x) \times D_{NS} \times A_i(x)\,dx + \int \beta_S(t,x) \times D_S \times A_i(x)\,dx$$

$$+ \int \beta_{RES}(t,x) \times D_{RES} \times A_i(x)\,dx + \int \beta_{LES}(t,x) \times D_{LES} \times A_i(x)\,dx$$

$$+ \gamma_{Age}(t) \times Age_i + \gamma_{Sex}(t) \times Sex_i + \gamma_{Ethnicity}(t) \times Ethnicity_i$$

$$+\gamma_{height}(t) \times height_i + \gamma_{Weigh}\ (t) \times Weight_i$$

$$+\gamma_{respiratory\ disease}\ (t) \times respiratory\ disease_i$$

$$+\gamma_{number\ of\ chron\quad diseases}(t) \times number\ of\ chronic\ diseases_i \qquad (2)$$

where $\mathrm{D_{NS}}, \mathrm{D_S}, \mathrm{D_{RES}}$ and $\mathrm{D_{LES}}$ are “dummy” binary variables indicating the belonging to the never smokers, smokers, recent ex-smokers (within 10 years), long-term ex-smokers (more than 10 years), respectively. In (2), all the continuous covariates (scalar or function) were centred to allow interpretation of the fitted coefficients. The functional coefficients $\alpha_{NS}(t)$, $\alpha_S(t)$, $\alpha_{RES}(t)$, $\alpha_{LES}(t)$, $\gamma_{Age}(t)$, $\gamma_{Sex}(t)$, $\gamma_{Ethnicity}(t)$, $\gamma_{height}(t)$, $\gamma_{Weigh}\ (t)$, $\gamma_{respiratory\ disease}\ (t)$ and $\gamma_{number\ of\ chronic\ diseases}(t)$ have the same interpretation as in the equation (1) apart that they are function of time, *t*, defined over the range between 0 and 5 seconds. For example, the intercept function $\alpha_{NS}(t)$ represents the expired volume over time among never smokers when all other predictors are at their centered value. In the same way, $\alpha_S\ (t)$, $\alpha_{RES}(t)$, and $\alpha_{LES}(t)$ corresponds to the intercept function for current smokers, recent ex-smokers and long-term ex-smokers, respectively.

The association between physical activity and respiratory function is assessed through the regression coefficient surfaces $\beta_{NS}(t,x)$, $\beta_S(t,x)$, $\beta_{RES}(t,x)$ and $\beta_{LES}(t,x)$. The surface $\beta_{NS}(t,x)$ illustrates the association between the diurnal accelerometry distribution with the expired volume-time profile among never smokers. The surfaces $\beta_S(t,x)$, $\beta_{RES}(t,x)$ and $\beta_{LES}(t,x)$ assess how the accelerometry and spirometry association is modified among current, recent or long-term ex-smokers respectively.

The model (2) also allows the inclusion of smoking status interaction terms with age, sex, ethnicity, height, weight, respiratory disease, and number of chronic diseases. Since this more complex model did not improve the function on function regression fit both in term of Akaike (AIC) and Bayesian (BIC) Information Criterion, we decided to restrict our analyses to the more parsimonious model (here model 2).

Supplementary Methods 6. Estimated change in the FEV_1 and FVC using parameters of the function on function regression model.

The function-to-function regression model described in Appendix Methods 6 allowed to identify in each smoking history group accelerometry intensity threshold, denoted x_0, above which physical activity is significantly associated with better lung function. Then, we estimated the association of an increment in 10 minutes spent above this threshold with FEV_1 and FVC as described below.

Let denote by $\beta(t,x)$ a surface coefficient for a given smoking status. For a fixed daily waking time T, an increase by a factor 1+r of the proportion of accelerometry values beyond the threshold x_0 results in a decrease in the proportion of accelerometry values. This will modify the initial daily accelerometry distribution function $A(x) = T \times f(x)$ to $\check{A}(x) = T \times \check{f}(x)$ with $\check{f}(x) = (1+r) \times f(x) \times \mathbb{1}_{x \geq x_0} + (1-s) \times f(x) \times \mathbb{1}_{x<x_0}$ (notations of **e-Appendix 3**).

The constant s is deduced based on the constraint of the function $\check{f}$ that is a density function with an integral equal to 1.

$$
\begin{aligned}
1 &= \int \check{f}(x)dx \\
&= \int (1+r) \times f(x) \times \mathbb{1}_{x \geq x_0} + (1-s) \times f(x) \times \mathbb{1}_{x<x_0} dx \\
&= (1+r) \times \int f(x) \times \mathbb{1}_{x \geq x_0} + (1-s) \times \int f(x) \times \mathbb{1}_{x<x_0} dx \\
&= (1+r) \times I_{x_0} + (1-s) \times \left(1 - I_{x_0}\right)
\end{aligned}
$$

where $I_{x_0} = \int_{x0}^{+\infty} f(x)\, dx$ and $\mathbb{1}_E$ denotes the indicator function of the interval E.

Finally, $1 = I_{x_0} + r \times I_{x_0} + \left(1 - I_{x_0}\right) - s \times \left(1 - I_{x_0}\right) \Rightarrow s = \frac{r \times I_{x_0}}{1 - I_{x_0}}$

So the corresponding daily accelerometry distribution function becomes:

$$\check{A}(x) = A(x) + \left(r \times \mathbb{1}_{x \geq x_0} - s \times \mathbb{1}_{x<x_0}\right) \times A(x)$$

All other covariates remaining equal, the change in the expired air volume-time curve is:

$$\Delta y(t) = \check{y}(t) - y(t)$$

$$= \int_0^{+\infty} \beta(t,x) \times \check{A}(x)dx - \int_0^{+\infty} \beta(t,x) \times A(x)dx$$

$$= \int \beta(t,x) \times \left(r \times \mathbb{1}_{x \geq x_0} - s \times \mathbb{1}_{x < x_0}\right) \times A(x)\, dx$$

$$= r \int_{x_0}^{+\infty} \beta(t,x) \times A(x)dx - s \int_0^{x_0} \beta(t,x) \times A(x)dx$$

Therefore, the quantities ΔFEV_1 and ΔFVC can then be calculated from $\Delta y(t)$ for t=1 second and t=5 seconds respectively.

To investigate the variability of $\Delta y(t)$, we postulate the normality of the maximum likelihood estimator of the $\beta(t,x)$ coefficients. More precisely, the surface $\beta(t,x)$ is defined by its coefficients $\left(\hat{\beta}\right)_i$ in basis expansion: $\beta(t,x) = \sum_{i=1}^{M} \hat{\beta}_i \times \mathbb{B}_i(t,x)$

where $\left(\mathbb{B}_i(t,x)\right)_{i=1,\dots,M}$ represents a two-dimensional basis (tensor splines for example).

Assuming a multivariate normal distribution with the estimated coefficient's covariance matrix Γ_β, we can sample a set of coefficients

$$\hat{\beta}^{(k)} = \left(\hat{\beta}_1{}^{(k)}, \dots, \hat{\beta}_M{}^{(k)}\right) \sim \mathcal{N}\left(\begin{pmatrix} \beta_1 \\ \\ \beta_M \end{pmatrix}, \Gamma_\beta\right), \text{ k=1..n}$$

and deduce a set of the surface coefficients:

$$\hat{\beta}_k(t,x) = \sum_{i=1}^{M} \hat{\beta}_i{}^{(k)} \times \mathbb{B}_i(t,x).$$

The variability of $\Delta y(t)$ is then assessed through the values $\left(\Delta y^{(k)}\right)_{k=1..n}$ where

$$\Delta y_{(k)}(t) = r \times I_1^{(k)} - s \times I_2^{(k)}$$

with $I_1 = \int_{x_0}^{+\infty} \beta^{(k)}(t,x) \times A(x)dx$ and $I_2 = \int_0^{x_0} \beta^{(k)}(t,x) \times A(x)dx$.

For a fixed value of t (1 second and 5 seconds for FEV_1 and FVC respectively), the variance (resp. confidence interval) of $\Delta y(t)$ can therefore be estimated by the variance (resp. the 2.5% and 97.5% percentiles) of the set of values $\left(\Delta y^{(k)}(t)\right)_{k=1..n}$ over n=10.000 simulations.

Supplementary Figure 1. Association of sex and age with expired air volume-time curve

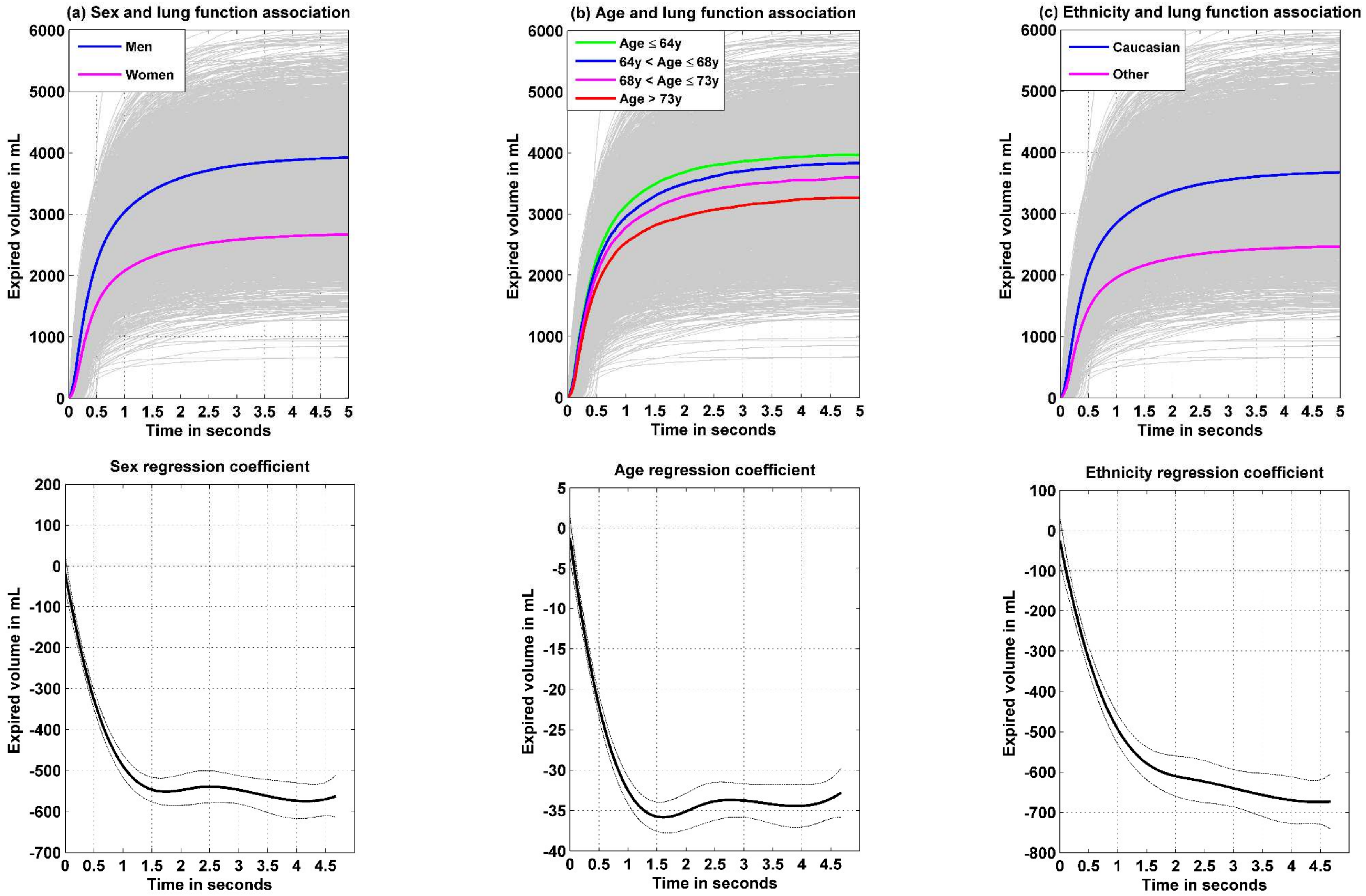

Supplementary Table 1. Association between physical activity and respiratory function excluding participants with respiratory diseases

	Total population	Smoking history			
		Current smokers	Recent ex-smokers	Long-term ex-smokers	Never smokers
	β* [95% CI]	β [95% CI]	β [95% CI]	β [95% CI]	β [95% CI]
**FVC (ml)**					
Model 1†					
For 10 min increase per day					
Sedentary behaviour	-2.5 [-5.0, -0.1]	-17.1 [-32.5, -1.8]	-7.6 [-16.9, 1.7]	-2.4 [-6.2, 1.4]	-1.4 [-4.7, 2.0]
Light activity	0.8 [-2.9, 4.4]	11.4 [-10.7, 33.6]	2.0 [-11.9, 16.0]	3.6 [-2.1, 9.2]	-2.0 [-6.9, 2.9]
MVPA	10.9 [4.9, 17.0]	53.6 [13.7, 91.5]	34.4 [10.3, 58.6]	14.8 [5.9, 23.7]	3.6 [-4.6, 11.7]
Model 2‡					
For 10 min increase per day					
Sedentary behaviour	-2.3 [-4.9, 0.3]	-16.5 [-31.8, -1.2]	-4.9 [-14.4, 4.5]	-2.4 [-6.3, 1.4]	-1.2 [-4.5, 2.2]
Light activity	0.4 [-3.2, 4.1]	9.1 [-13.2, 31.3]	-0.4 [-14.4, 13.6]	3.3 [-2.4, 8.9]	-2.2 [-7.1, 2.7]
MVPA	10.3 [4.1, 16.4]	50.3 [11.4, 89.3]	27.4 [2.7, 52.0]	14.2 [5.3, 23.2]	3.1 [-5.1, 11.3]
**FEV_1 (ml)**					
Model 1†					
For 10 min increase per day					
Sedentary behaviour	-1.1 [-3.2, 1.6]	-11.4 [-24.3, 1.6]	-6.3 [-14.2, 1.5]	-1.0 [-4.2, 2.2]	-0.1 [-2.9, 2.7]
Light activity	-0.3 [-3.3, 2.8]	6.8 [-11.9, 25.5]	4.8 [-7.1, 16.6]	1.4 [-3.3, 6.2]	-2.5 [-6.6, 1.7]
MVPA	6.6 [1.5, 11.8]	24.1 [-8.8, 57.0]	26.1 [5.6, 46.5]	10.3 [2.8, 17.8]	0.8 [-6.1, 7.7]
Model 2‡					
For 10 min increase per day					
Sedentary behaviour	-0.6 [-2.8, 1.5]	-10.7 [-23.6, 2.3]	-4.3 [-12.3, 3.7]	-0.7 [-4.0, 2.6]	0.3 [-2.5, 3.1]
Light activity	-0.9 [-4.0, 2.2]	3.2 [-15.6, 22.0]	2.9 [-9.0, 14.7]	0.8 [-4.0, 5.6]	-2.9 [-7.0, 1.3]
MVPA	5.8 [0.6, 11.0]	21.5 [-11.4, 54.4]	21.1 [0.2, 42.0]	9.4 [1.8, 16.9]	0.3 [-6.6, 7.2]

MVPA: Moderate to vigorous physical activity. FEV_1: Forced Expiratory Volume in 1 sec. FVC: Forced Vital Capacity. CI: confidence interval.
*Additional adjustment on smoking history. †Model 1: adjusted on age, sex, ethnicity, height, weight, and waking duration. ‡Model 2: Model 1 additionally adjusted on occupational position at age 50y, education, marital status, alcohol consumption, fruit and vegetable consumption, and number of chronic diseases. Model 2 was additionally adjusted for number of cigarettes smoked per day for the current smokers and the recent ex-smokers (corresponding to cigarettes smoked before they quitted smoking).
